# Effect of estradiol valerate on humoral immune function in BALB/c mice after total hysterectomy

**DOI:** 10.1590/1414-431X2025e14651

**Published:** 2025-06-20

**Authors:** Penghuan Chang, Hui Huang, Peijian Huang, Hongru Yi, Wenhua Zhong, Zhaoxin Yang

**Affiliations:** 1 Department of Nursing, Haikou Affiliated Hospital of Central South University, Xiangya School of Medicine, Haikou, China; 2 College of Pharmacy, Hainan Medical University, Haikou, China; 3 Department of Neurology, Hainan General Hospital, Hainan Hospital Affiliated to Hainan Medical University, Haikou, China; 4 Department of Pharmacy, Guangdong Provincial Hospital of Chinese Medicine, Hainan Hospital, Haikou, China; 5 Research Center for Drug Safety Evaluation of Hainan Province, Hainan Pharmaceutical Research and Development Science Park, Hainan Medical University, Haikou, China

**Keywords:** Total hysterectomy, Estrogen, T-cell-dependent antibody response (TDAR), Immune function, B lymphocytes

## Abstract

Total hysterectomy is a commonly performed gynecological procedure used to treat uterine pathologies. This study aimed to establish a total hysterectomy mouse model and investigate the effect of estradiol valerate on humoral immune function in BALB/c mice. Our results showed that the ratios of CD4^+^ and CD8^+^ subpopulation and hepatitis B virus antibody IgG content were reduced in the total hysterectomy (TH) group compared with the control group (P<0.05). The measured indices showed varying degrees of improvement after estradiol valerate administration. The numbers of CD19^+^ B lymphocytes significantly increased in the TH group (P<0.05) compared with the control group. There was a significant reduction of the size of germinal centers within lymph nodes in the TH group. This effect was reversed by estrogen supplementation. In summary, reduced estrogen levels following total hysterectomy may impair B lymphocyte activation, IgG secretion, and plasma cell production, leading to compromised immune function. Early treatment with estradiol valerate reverses impaired immune function and enhances antigen-induced antibody production in mice.

## Introduction

Total hysterectomy is a commonly performed gynecological procedure for treating uterine pathologies (1). It is primarily used to treat conditions such as uterine smooth muscle tumors, endometriosis, abnormal uterine bleeding, uterine adenomatosis, reproductive tract prolapse, and cancers of the uterus or other reproductive organs. The uterus is a vital reproductive organ that also functions as an endocrine organ with complex roles. Hormones, cytokines, enzymes, and other biologically active substances released by the uterus regulate both local and systemic physiological and pathological processes. These uterine factors are also vital in maintaining and conserving the dynamic equilibrium of the organism (2). The removal of the uterus disrupts the anatomical and functional interaction between the ovaries and the uterus, leading to reduced ovarian function and a decline in estrogen levels. Generally, hysterectomy damages the ovarian branch of the uterine artery, resulting in a 50-70% reduction in the blood supply to the ovaries. This damage affects ovarian blood flow and function, leading to a significant decrease in serum estrogen levels. As there is no gradual adaptation period, the abrupt cessation of hormone production leads to a significant exacerbation of menopausal-like symptoms (3).

Estrogen plays a key role in regulating the proliferation, differentiation, and activation of various immune cells. The estrogen receptor (ER) is present in various immune cells, such as B and T cells, indicating that estrogen has a potential regulatory role in immune cell function (4,5). The intracellular effects of estrogen, mediated through both genomic and non-genomic pathways, are exerted via specific intracellular receptors, namely ERα and Erβ, and membrane receptors. The ERα is predominantly expressed in CD4^+^T cells, while ERβ is present mainly in B cells. CD8^+^T cells express both ERα and ERβ. Estrogen has been shown to regulate different T-cell subsets, including CD4^+^and CD8^+^ cells (6), and engages with the membrane-bound G protein-coupled estrogen receptor GPER to regulate T-cell-mediated immune responses (7). Estrogen enhances the population of bone marrow progenitor B cells and the viability of spleen B lymphocytes and promotes the maturation of autoreactive B cells (8). Studies have consistently demonstrated the significant role of estrogen in regulating peripheral immune functions.

Total hysterectomy may lead to a postoperative decline in ovarian function and estrogen levels, both of which are crucial for regulating the immune function. It remains unclear whether early hormone replacement therapy may reverse postoperative alterations in immune function. Therefore, the present study was designed to investigate the impact of early estradiol replacement in a total hysterectomy BALB/c mice model, with the hepatitis B vaccine serving as the antigen for stimulation of the immune response.

## Material and Methods

### Animals

BALB/c female mice (6-8 weeks, 16-18 g body weight) were obtained from the Guangzhou Medical Laboratory Animal Center (License SCXK(GD) 2022-0002). All mice were housed in a controlled environment with specific pathogen-free conditions, with temperatures ranging from 22-25°C, relative humidity between 45-60%, and a 12-h light/dark cycle. Throughout the experimental period, standard rodent food and filtered tap water were provided *ad libitum*. The animal care and treatment protocols in this study were approved by the Ethics Committee of Hainan Medical University (HYLL-2021-148).

### Research design

**Figure 1:**
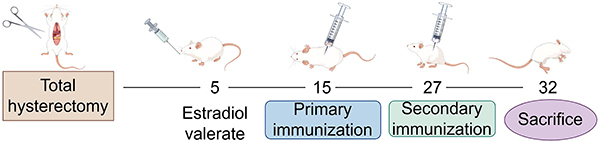
Schematic of the research design. In brief, the mice were treated with estrogen on the 5th day after total hysterectomy, the first immunization was performed on the 15th day, the second immunization was performed on the 27th day, and the mice were sacrificed on the 32nd day. By FigDraw (http://www.figdraw.com).

Twenty-four mice were divided into three groups (n=8 per group): Control group, total hysterectomy group (TH), and total hysterectomy + estradiol valerate group (TH+EV). On the 5th day post-surgery, the control and TH groups were administered sterilized water orally, while the TH+EV group received of estradiol valerate (0.1 mL,0.04 mg/mL; Bayer HealthCare Co., Ltd., China) via oral gavage for 28 consecutive days. For the T-cell-dependent antibody response (TDAR) test, hepatitis B vaccine (1 mL, 0.246 μg/mL, North China Pharmaceutical Jintan Biotechnology Co., China) was subcutaneously injected on days 15 and 27 after total hysterectomy. After 24 h of the last hepatitis B vaccine injection, the mice were anesthetized with isoflurane, blood samples were collected from the inner canthus vein, and mice were euthanized. The ovaries, spleen, lymphatic system, thymus, and brain were removed and weighed. All organs except the brain were subsequently immersed in a 4% neutral formaldehyde solution for fixation. The data obtained from these organs were used to compute the visceral organ-brain ratio. Blood and serum samples were collected for subsequent experimentation (Figure 1). The body weights of mice were measured every other day throughout the testing period to monitor any fluctuations. No treatments, such as food deprivation, were administered prior to weighing the mice.

### Total hysterectomy in mice

Mice were fasted without water for 12 h prior to the surgical procedure. Gaseous anesthesia using isoflurane (MatrxVIP3000 gas anesthesia system, Matrx, USA) was continuously administered to the experimental animals positioned in a supine posture. The abdominal skin was disinfected following standard procedures, and a transverse incision of approximately 0.5 cm was made at the midpoint of the lower abdomen to access the uterus. The right and left lateral bicornuate uterus were separated. The uterus was incised at the junction between the uterine horn and the ovary and then completely detached, avoiding any damage to the uterus, ovary, and the associated blood vessels. The uterus was then ligated using a 0-1 surgical thread at the junction between the cervix and the vagina. Subsequently, the uterus was clamped above the site of cervical ligation, followed by closure of the abdominal cavity and suturing and sterilization of the skin. After surgery, the animals were provided with sufficient feed and drinking water.

### Calculation of the organ-brain ratios

At the conclusion of the experiment, the spleen, thymus, and brain were dissected and weighed, and each organ coefficient was calculated using the following formula: organ-brain ratio (mg/mg) = organ weight / brain weight × 100%.

### Histopathological examination

The spleen, ovary, and lymph node tissues were preserved for 48 h in neutral buffered 4% paraformaldehyde and then embedded in paraffin for further processing. Tissue slices approximately 3-µm thick were sectioned and mounted on slides. After hematoxylin and eosin staining, pathological changes in the tissue were observed under a light microscope.

### Analysis of lymphocyte subsets by flow cytometry

Peripheral blood samples were incubated with the following antibodies from BD Biosciences (BD, USA): CD3-peridinin-chlorophyll-protein (CD3-PerCP), CD19-fluorescein-isothiocyanate (CD-19-FITC), CD8-phycoerythrin (PE), and CD4-allophycocyanin (CD4-APC), followed by resuspension in phosphate-buffered saline. The samples were analyzed using a flow cytometer, model C6 (BD Bioscience).

### TDAR test and analysis

The TDAR test was used to assess the production of antibodies specific to the hepatitis B virus following hysterectomy. A fully automated chemiluminescence method was used to determine surface antibody levels for the hepatitis B virus in peripheral blood. Serum samples were mixed with a particulate magnetic suspension and enzyme conjugate, incubated at an elevated temperature, and then washed five times with a washing solution. Substrates A and B were added, and antibody concentrations were determined using the AutoLumo A2000 Plus apparatus (Zhengzhou Antu Bioengineering Co., Ltd., China) and calibration curve.

### Immunofluorescence staining

The tissues were stained using multiplex immunofluorescence with the tyramide signal amplification technique. The spleen and lymph nodes were embedded in paraffin, dehydrated, and then incubated with primary antibodies: CD19 polyclonal antibody (1:200) and CD138/syndecan-1 polyclonal antibody (1:1000), both from Proteintech (BD, USA). Samples were then incubated with secondary antibodies, goat anti-rabbit IgG H&L (Cy3^®^) preadsorbed (1:200; Abcam, China), and goat anti-rabbit IgG H&L (Alexa Fluor^®^ 488, 1:500; Abcam, China) at room temperature protected from light. Samples were then treated with an anti-fluorescence quenching blocking agent (Wuhan Bolf Biotechnology Co., Ltd., China) and examined under a laser confocal fluorescence microscope (OLYMPUS, Japan).

### Statistical analysis

All data were analyzed using SPSS version 25.0 software (IBM, USA). Data are reported as means±SD. All graphs in this study were prepared using GraphPad Prism 9.5 software (USA). Multiple sets of data were compared with one-way analysis of variance. P<0.05 was considered statistically significant.

## Results

### Body weight curve

**Figure 2:**
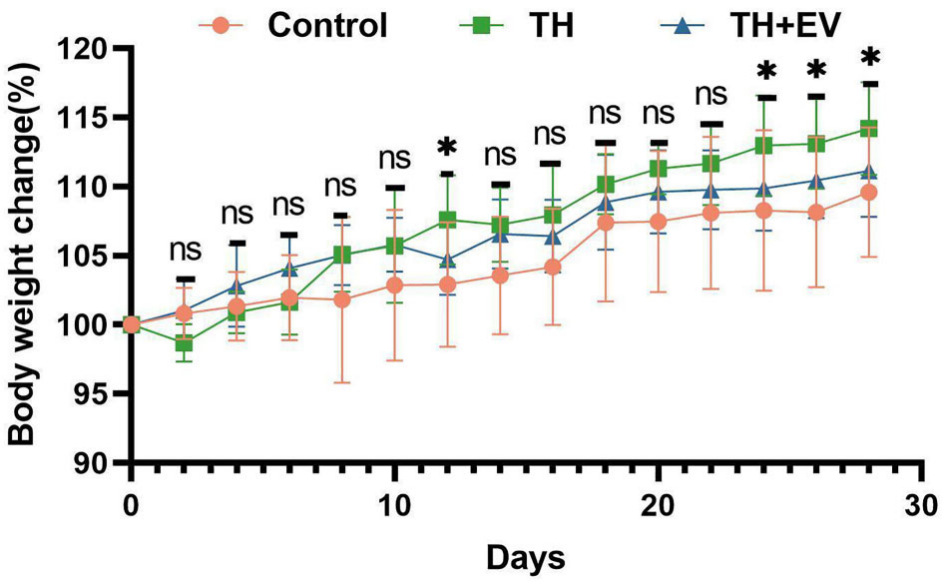
Percentage of body weight change in each experimental group of mice. Data are reported as means and SD (n=8/group). *P<0.05, Control group compared with TH group (ANOVA). ns: non-significant; TH: total hysterectomy; EV: estradiol valerate.

After 28 days of treatment, body weights increased in all groups (Figure 2). However, the weight gain was significantly higher in the TH group than in the control group (P<0.05, control 22.85±1.50 g, TH 24.53±1.33 g). From day 12, the body weight of the TH+EV group began to converge with that of the control group following estrogen administration, and by day 28, the two groups were equivalent. These findings indicated that total hysterectomy resulted in an increased body weight, a change that appears to be associated with a reduction in estrogen levels. Estrogen plays an important role in promoting the metabolism of fat and sugar in females, and its deficiency may lead to water and sodium retention and fat accumulation.

### Spleen and thymus organ-brain ratios

**Figure 3:**
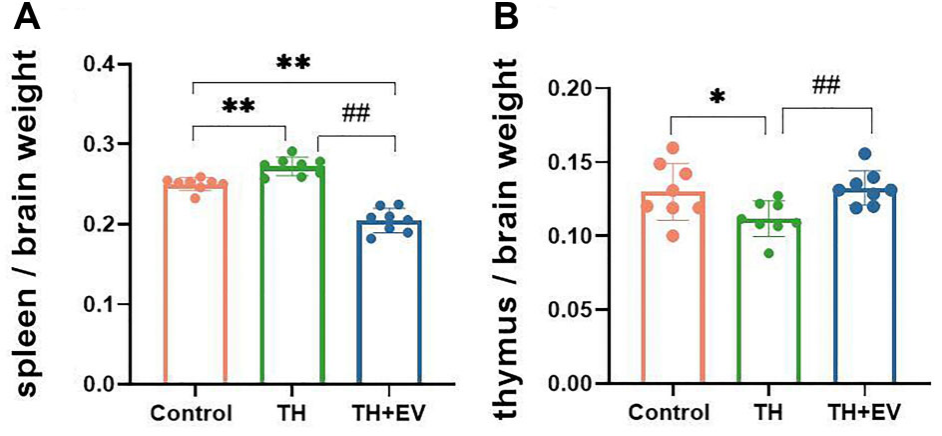
Effects of estrogen on immune organs spleen and thymus. **A**, Spleen/brain weight analysis results. **B**, Thymus/brain weight analysis results. Data are reported as means and SD (n=8/group). *P<0.05, **P<0.01, ^##^P<0.01 (ANOVA). TH: total hysterectomy; EV: estradiol valerate.

The spleen-brain ratio was significantly higher in the TH group than in the control group (P<0.01, control 0.25±0.01, TH 0.27±0.01), suggesting that the spleen in the TH group displayed an increase in mass after being stimulated by an antigen. This phenomenon may be closely related to estrogen deficiency and the body’s compensatory response, as demonstrated by a mass reduction in the spleen upon estrogen supplementation (P<0.01 *vs* TH group, TH+EV 0.20±0.02). A possible reason for the altered spleen size is that after being stimulated by antigens, the body attempts to produce more antibodies through the transformation of plasma cells. However, due to the relatively low level of estrogen in the TH group, the transformation of B cells into plasma cells is hindered, resulting in an increase in spleen mass. However, the thymus-brain ratio in the TH group was lower than that in the control group (P<0.05, control 0.13±0.02, TH 0.11±0.01). Exogenous estradiol valerate treatment restored the thymus-brain ratio of the TH mice to normal level (P<0.01 *vs* TH group, TH+EV 0.13±0.01) (Figure 3), suggesting that estrogen may also be involved in the differentiation and proliferation of thymic T lymphocytes.

### Histomorphological changes of the spleen, lymph nodes, and ovaries

**Figure 4:**
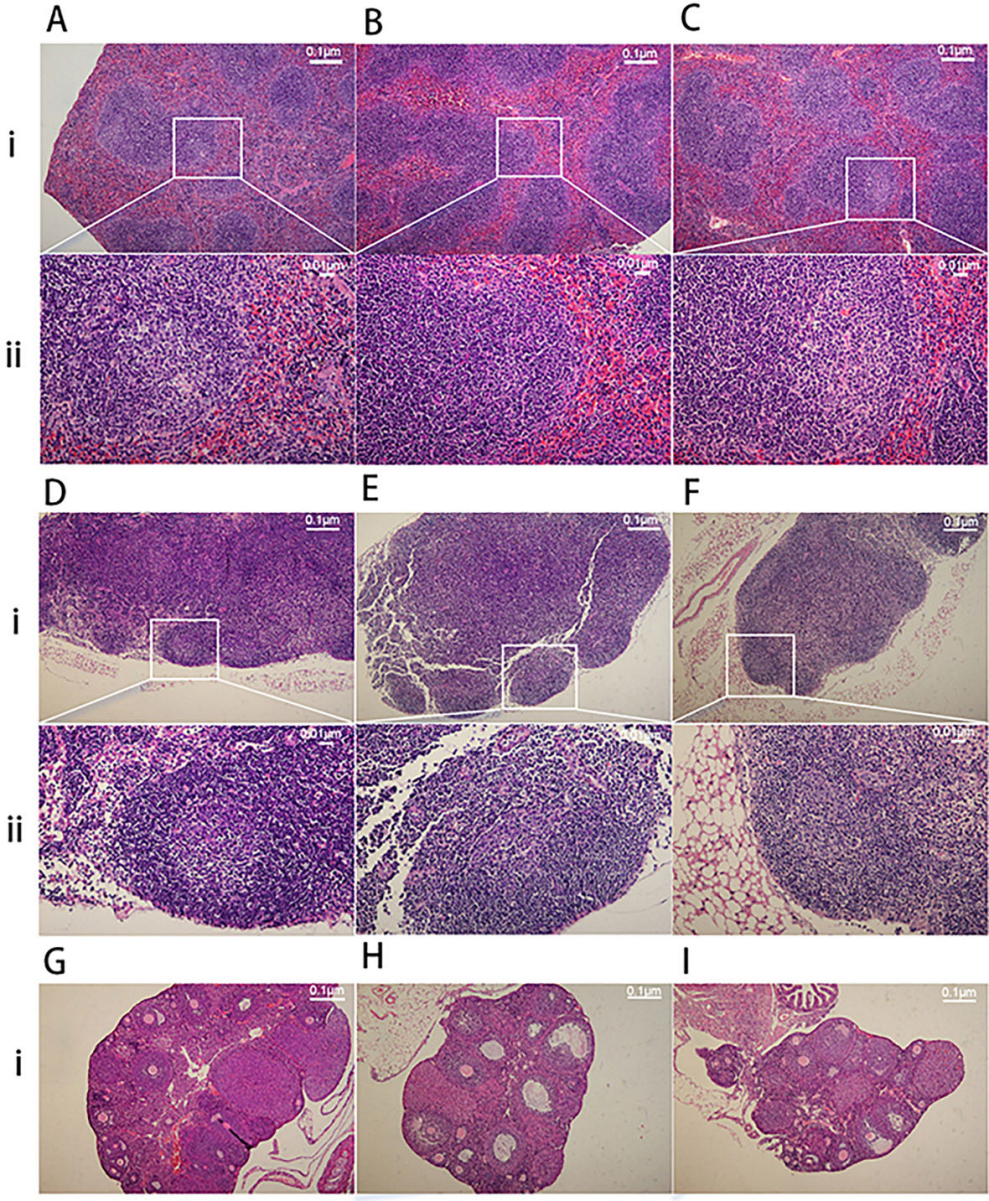
Hematoxylin and eosin staining of the morphological structure of spleen, lymph nodes, and ovarian tissues. **A**, **i**: Spleen in Control group, **ii**: Partial enlargement of Figure **A**-**i**. **B**, **i**: Spleen in the TH group, **ii**: Partial enlargement of Figure **B**-**i**. **C**, **i**: Spleen in the TH+EV group, **ii**: Partial enlargement of Figure **C**-**i**. **D**, **i**: Lymph nodes in the control group, **ii**: Partial enlargement of Figure **D**-**i**. **E**, **i**: Lymph nodes in the TH group, **ii**: Partial enlargement of Figure **E**-**i**. **F**, **i**: Lymph nodes in the TH+EV group, **F**, **ii**: Partial enlargement of Figure **F**-**i**. **G**, Spleen in the control group. **H**, Spleen in the TH group. **I**, Spleen in the TH+EV group. TH: total hysterectomy; EV: estradiol valerate. Scale bars 0.1 and 0.01 μm.

Hematoxylin and eosin staining indicated that the spleen in the control group exhibited an enlargement of the germinal centers and an increased number of B lymphocytes in node growth centers. Under normal conditions, when stimulated by antigen to cause a humoral immune response, the splenic lymphoid nodules increase in size. There was a significant reduction of germinal center size within lymph nodes in the TH group and a less pronounced change in the TH+EV group (Figure 4A-C). Concurrently, lymph nodes also showed a similar change in size (Figure 4D-F), suggesting that estrogen plays a role in humoral immunity and B cell activation. After total hysterectomy, the ovaries of mice in the TH group were reduced in size, had a decreased follicle count, and exhibited hypofunction. These conditions showed no significant improvement after estrogen administration (Figure 4G-I).

### Effect of estrogen on B lymphocytes in humoral immunity

**Figure 5:**
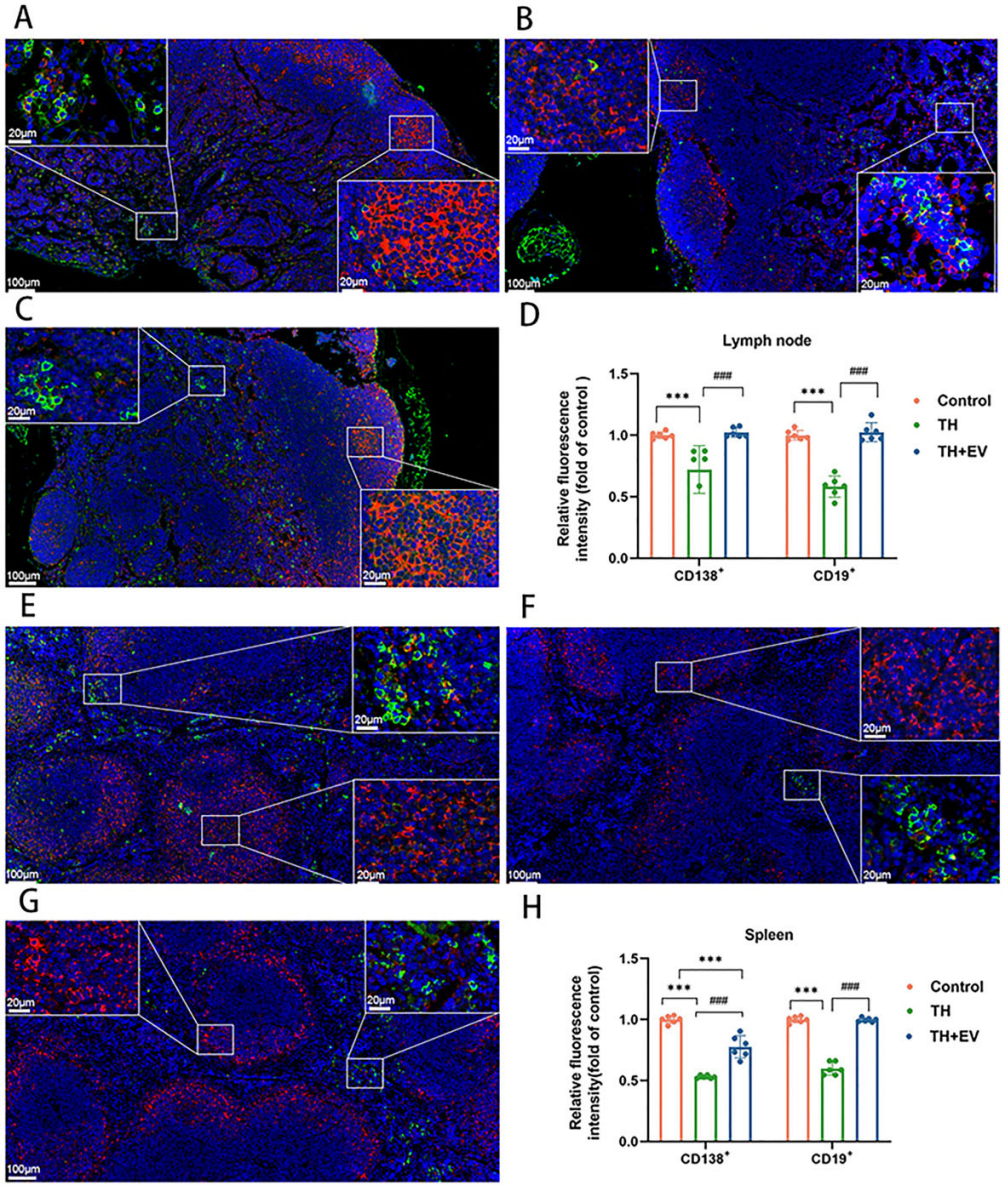
Immunofluorescence staining analysis of the effect of estrogen on B cells and plasma cells in mice. **A**-**C**, Expression of CD19^+^ and CD138^+^ positive cells in lymph nodes of the Control, TH, and TH+EV groups. **D**, Quantitative analysis of CD19^+^ and CD138^+^ positive cell expression levels in lymph nodes. **E**-**G**, Expression of CD19^+^ and CD138^+^ positive cells in the spleen of the control, TH, and TH+EV groups. **H**, Quantitative analysis of CD19^+^ and CD138^+^ positive cell expression levels in the spleen. Data are reported as means and SD (n=8/group). ***P<0.01, ^###^P<0.01 (ANOVA). TH: total hysterectomy; EV: estradiol valerate. Scale bars 100 and 20 μm.

In the experimental mice, lymphoid nodules of splenic follicles and lymph nodes, as well as some spleen cords and lymphatic medullary cords, primarily contained CD19^+^ B lymphocytes (Figure 5). The CD138^+^ plasma cells were predominantly located in the splenic cords of the spleen and lymphatic medulla. Immunofluorescence staining showed that the levels of CD19^+^ B lymphocytes and CD138^+^ plasma cells in the lymph nodes were significantly lower in the TH group versus the control group. Following estrogen intervention, an increase in the presence of these two cell types was observed within the lymph nodes of the TH+EV group (Figure 5A-D). Similar changes were observed in the spleen (Figure 5E-H).

### Effect of estrogen on lymphocyte differentiation and proliferation

**Figure 6:**
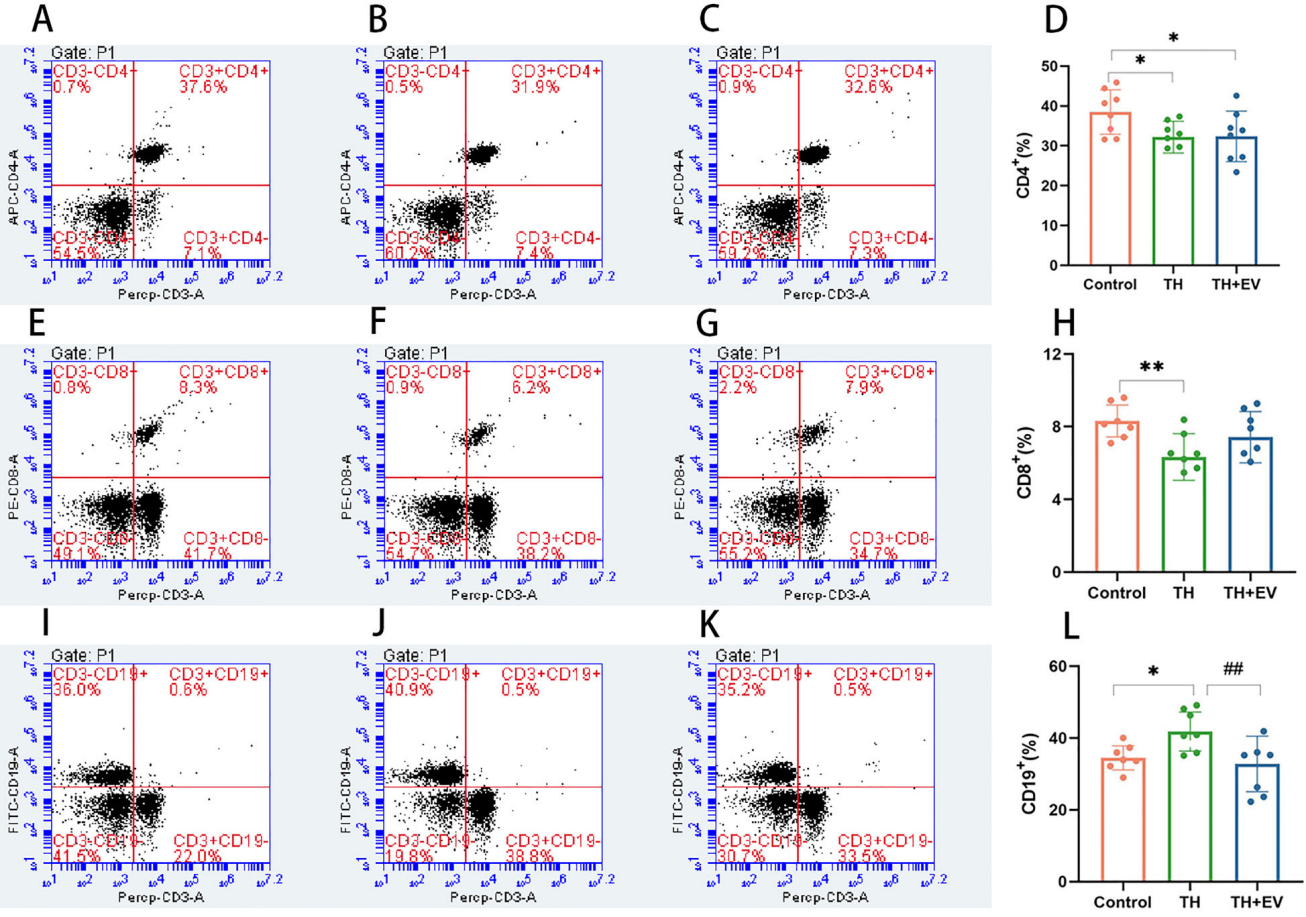
Effects of estrogen on CD4^+^, CD8^+^, and CD19^+^ lymphocyte subsets in peripheral blood of mice analyzed by flow cytometry. Percentage of CD4^+^ cell subsets in the peripheral blood of mice in the control (**A**), TH (**B**), and TH+EV groups (**C**); **D**, Statistical analysis of the percentage of CD4^+^ cells. Percentage of CD8^+^ cell subsets in the peripheral blood of the control (**E**), TH (**F**), and TH+EV groups (**G**); **H**, Statistical analysis of the percentage of CD8^+^ cells. Percentage of CD19^+^ cell subsets in the peripheral blood of the control (**I**), TH (**J**), and TH+EV groups (**K**); **L**, Statistical analysis of the percentage of CD8^+^ cells. Data are reported as means and SD (n=8/group). *P<0.05, **P<0.01, ^##^P<0.01 (ANOVA). TH: total hysterectomy; EV: estradiol valerate.

Compared with the control group, the TH group showed a decrease in the levels of CD4^+^ and CD8^+^ cells (P<0.05). Following estrogen administration, both cell types showed a trend toward recovery (CD4, P>0.05 *vs* TH group, TH 32.17±3.96, TH+EV 32.35±6.32; CD8, P>0.05 *vs* TH group, TH 6.33±1.28, TH+EV 7.43±1.41). Although the statistical analysis did not reveal any significant differences, these findings suggest the potential involvement of estrogen in the differentiation and proliferation of T lymphocytes. In the TH group with estrogen deficiency, T lymphocyte differentiation and proliferation were similar to those observed in the antigen-stimulated TH group. This effect was reduced following estrogen administration. The phenomenon observed in the control group was alleviated with estrogen supplementation. The changes in peripheral blood CD19^+^B cells differed from those in T lymphocytes, with significantly greater alterations in the TH group compared to the control group (P<0.05). However, after estrogen supplementation, the levels of CD19^+^B cells in the TH+EV group were similar to those in the control group. These results suggest the potential involvement of estrogen in the activation of B lymphocytes. When estrogen levels decreased, the body responded to antigenic stimulation by initiating compensatory mechanisms, such as increased B cell proliferation (Figure 6A-L).

### Estrogen contributed to the production of specific antibodies

**Figure 7:**
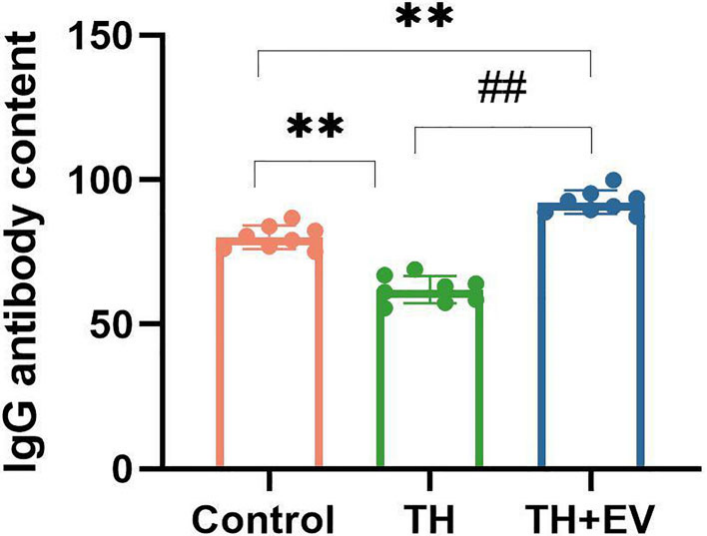
The content of IgG in serum was analyzed by the T-cell-dependent antibody response (TDAR) experiment. Mice with total hysterectomy were treated with estradiol for 28 days after hepatitis B virus stimulation. Peripheral serum was collected to detect the level of hepatitis B virus surface specific antibody IgG by automatic chemiluminescence assay. Data are reported as means and SD (n=8/group). **P<0.01, ^##^P<0.01 (ANOVA). TH: total hysterectomy; EV: estradiol valerate.

Compared with the control group, the concentration of IgG antibodies was lower in the TH group (P<0.01). Following estradiol valerate administration, the levels of IgG antibodies were significantly elevated in the TH+EV (P<0.01) compared with the TH group. Furthermore, estrogen resulted in the recovery of humoral immune function and promoted the generation of specific antibodies to modulate the immune response (Figure 7). Thus, estrogen may positively impact humoral immune function in mice.

## Discussion

The present study determined that short-term estrogen supplementation also affects immune function disrupted by hysterectomy in mice. Estradiol valerate administration resulted in a significant increase in hepatitis B surface antibodies in the peripheral blood of hepatitis B-vaccinated mice after hysterectomy. This suggests that estrogen may positively influence the humoral immune function in mice following total hysterectomy. This intervention may help restore humoral immune function and promote the generation of specific antibodies to regulate the immune response. A previous study reported that estrogen significantly enhanced antibody activity by affecting the glycosylation of the IgG-Fc region (9), suggesting that estrogen stimulates antibody production, antibody activity, and maturation. Thus, estrogen can function as an endogenous regulator of IgG and potentially modulate immune function.

In the present study, mice that underwent total hysterectomy exhibited an increase in postoperative body weight. Estrogen supplementation affected the change in body weight, suggesting a potential correlation between increased weight and decreased estrogen levels. A rapid increase in weight is commonly observed in humans during the perimenstrual phase, which is associated with decreased estrogen levels and changes in fat metabolism. Estrogen deficiency expedites the buildup of visceral fat, increasing the susceptibility to metabolic syndrome and potentially culminating in obesity (10,11). Obesity is a chronic inflammatory condition that enhances susceptibility to a variety of inflammatory disorders. The effect of obesity on the immune system correlates with the secretion of molecules by adipocytes and systemic pro-inflammatory traits (12).

The current study suggested that estrogen is involved in T lymphocyte differentiation and proliferation. In the TH group with relative estrogen deficiency, there was a notable reduction in the percentage of CD4^+^ and CD8^+^ lymphocytes in the peripheral blood following antigen stimulation compared with the control group. This reduction was reversed after estrogen supplementation, and known mechanisms of estrogen action on T lymphocytes likely account for the observed reduction in the thymus-brain organ coefficient in the TH group. Specifically, reduced estradiol activity likely led to the inhibition of T lymphocyte differentiation and proliferation, resulting in significantly lower organ coefficient levels in TH mice compared with controls. Notably, these pathophysiological changes were effectively reversed following therapeutic intervention. As a steroid hormone, estrogen plays a pivotal role in modulating physiological processes across multiple systems, including critical regulatory functions in the reproductive system through well-characterized endocrine pathways (13), as well as significant immunomodulatory effects. The latter actions primarily target specific immune cell subsets through dual mechanisms: genomic pathways mediated by ERs and rapid non-genomic signaling cascades (14,15). Estrogen regulates all T cell subsets, including CD4^+^ and CD8^+^T cells (16). In general, the effects of estrogen are thought to be mediated by nuclear ERα and ERβ, and ERα/ERβ can regulate CD4^+^T and CD8^+^T cell immunity (17). The CD4^+^ cells in T lymphocytes have higher ERα levels compared with ERβ, and the expressions levels of ERα and ERβ are both lower in CD8^+^ cells, a difference that may provide the mechanism through which estrogen regulates CD4^+^ and CD8^+^T cell differentiation (18). Estrogen can induce the functional changes in T lymphocytes by regulating the growth, differentiation, and cytokine production of CD4^+^T and CD8^+^T lymphocytes.

In the present study, the observed alteration in the B lymphocyte subpopulation of hysterectomized mice contrasted with the changes in T lymphocytes. After total hysterectomy, mice exhibited an increase in peripheral blood B cell counts when re-stimulated with exogenous antigens. In contrast, the number of CD19^+^ B cells was similar in hysterectomized and control mice following estrogen supplementation. This change was also observed in the spleen-brain organ coefficient. Specifically, compared with control groups, the spleen-brain organ coefficient significantly increased in the TH group, most likely because of the proliferation of B lymphocytes, and those changes were effectively reversed following therapeutic intervention. These findings suggest a potential role of estrogen in the mechanism of B lymphocyte activation. As estrogen levels decreased, it is possible that the body mounted compensatory responses to antigenic stimulation, including increased B cell proliferation. The abovementioned effect was reversed by estrogen supplementation, suggesting that a decline in estrogen levels may have impeded the differentiation of B cells into plasma cells.

Histomorphometric analysis revealed that the mice subjected to total hysterectomy exhibited a reduction of germinal center size within lymph nodes in the spleen following antigenic stimulation compared with control mice. The lymph nodes showed similar changes; however, this effect was reversed by estrogen supplementation. Fluorescent immunostaining of B cells (CD19^+^) and plasma cells (CD138^+^) revealed a significant reduction in plasma cells in the lymph nodes of total hysterectomized mice compared with controls. Previous research showed that the number of plasmablasts and IgM-secreting plasma cells were augmented in B cell cultures from both male and female animals following a 6-day treatment with pokeweed mitogen and estradiol (19). A study using rhesus monkeys showed that the peak levels of estrogen before ovulation led to an increase in the proportion of B cells that differentiate into IgG antibody-secreting cells (20). Another study showed that both cyclic and continuous treatments with estrogen led to increased production of anti-DNP IgG following DNP-KLH immunization, indicating that estrogen enhances antigen-specific antibody responses after an antigenic challenge (21). Estrogen also boosts the proliferation of B cells, prevents apoptosis, and supports the survival of autoreactive B cells through enhanced expression of BAFF, Bcl-2, CD22, and SH2. These factors, which include protein tyrosine phosphatase (SHP)-2, act as survival regulators (22). As indicated in various studies, reduced estrogen levels may hinder B lymphocyte activation and IgG secretion, thereby affecting plasma cell production.

Following total hysterectomy in BALB/c mice, estrogen levels decreased, leading to reduced immune function, which was subsequently improved with estradiol valerate administration. Thus, prompt administration of estradiol valerate effectively reversed the compromised immune function and enhanced antigen-induced antibody production. These findings indicated that estradiol treatment contributed to improved overall immune function.
